# Stigma and barriers to seeking mental health care among emergency department providers – A mixed methods study

**DOI:** 10.1371/journal.pmen.0000489

**Published:** 2025-11-20

**Authors:** Shannon C. White, Charlotte H. Harris, Ravindra Gopaul, Joshua M. Smyth

**Affiliations:** 1 Department of Psychology, The Ohio State University, Columbus, Ohio, United States of America; 2 Sidney Kimmel Medical College, Thomas Jefferson University, Philadelphia, Pennsylvania, United States of America; 3 Department of Emergency Medicine, Penn State Hershey Medical Center, Hershey, Pennsylvania, United States of America; National Psychological Association of Ukraine, UKRAINE

## Abstract

High levels of psychological distress and depression are common among clinicians working in US emergency departments [ED], yet treatment-seeking remains rare, contributing to burnout and negative well-being. Reluctance to seek care often reflects concerns about negative consequences (e.g., stigma, negative professional impact). We sought to characterize the availability of mental health resource availability, beliefs about seeking care, and barriers faced by emergency department healthcare providers. A sequential mixed-method design was employed. Phase 1 included a survey of ED physicians, advanced practice providers (APPs; i.e., nurse practitioners and physician assistants) and medical residents at an academic medical center. The survey assessed demographics, perceptions of support across occupational levels (e.g., institutional, unit/ department, direct supervisor, and peers), and workplace stressors. Phase 2 comprised qualitative interviews to explore survey findings in depth. The survey respondent sample (n = 43; 39% of all ED staff) included 20 physicians, 10 APPs, and 13 residents. Overall, 25.59% scored in the moderate to severe depression range on the PHQ-9. Self-stigma was moderate and significantly higher among males (M = 2.81, SD = 0.54) when compared to female (M = 2.19, SD = 0.52) providers. Common barriers to seeking care included lack of time (72%), privacy concerns (49%), worry about negative impact on licensing (37%), and expectation that treatment would not help (37%). Interviews (n = 16; 88% had completed the survey) reinforced these findings, identifying three commonly endorsed beliefs about 1) the need for occupationally tailored mental health resources, 2) the continued presence and perpetuation of mental health stigma, and 3) barriers to seeking mental health services. In addition to time constraints, many clinicians reported self-stigmatizing beliefs and/or worries about professional repercussions for seeking care for mental health issues. These data underscore pervasive impediments to seeking help in ED, which may negatively influence clinician well-being and performance over time.

## Background

In the early 2000’s, the National Academy of Medicine (formerly the Institute of Medicine) published reports suggesting that up to 100,000 people die annually in the United States because of medical error or preventable adverse events. As a result, organizations were forced to drastically change care delivery practices that would in turn increase occupational stress and burnout among providers. [1] Over the next two decades, healthcare organizations and clinicians implemented corrective actions to improve patient care, population health, and reduce costs. [1] The requirement to address these challenges effectively and deliver high-quality, patient-centered care depends in large part on the functioning of the clinical workforce. However, there is a consensus among system experts that because of occupational stress, mental health and well-being among clinicians, clinical students, and trainees is deteriorating. [2–6] Persistent exposure to high stress environments can lead to clinicians experiencing distress and/ or decreased mental quality of life (QOL), leading to potentially negative implications for patients. For instance, after controlling for other personal and professional factors, both depression and burnout remained independent predictors of recent major medical errors. [7,8]

Recently, progress has been made in addressing provider mental health and well-being. This includes the release of the National Academy of Medicine’s (NAM) release of the National Plan for Health Workforce Well-Being, the passing of the Dr. Lorna Breen Health Care Provider Protection Act (a bill that established grants to be awarded to hospitals, medical professional associations, and other health care entities to improve mental and behavioral health among healthcare providers), and the launch of the Action Collaborative on Clinician Well-Being and Resilience from the NAM. However, pervasive stigma remains among healthcare providers regarding seeking mental health treatment. Beyond the widespread societal stigmas about seeking mental health treatment that exists generally, stigma surrounding mental illness is believed to be more prevalent among healthcare clinicians and trainees than in the general population. [9–12] Stigma associated with seeking care is exacerbated by multiple factors. For instance, problematic licensure questions on initial and renewal applications for physicians and physician assistants (e.g., questions asking about mental health treatment sought across the lifespan versus in the previous 6 or 12 months) that in turn foster perceptions and beliefs about negative consequences of seeking mental health care both from state licensing boards and employers, [13,14] In addition, institutional barriers (e.g., types of services offered), job scheduling demands, and resource accessibility have also been shown to exacerbate mental health care related stigma. [13,14] Despite the growing body of research documenting barriers to seeking mental health care many specifics, such as barriers within individual specialties, are still poorly understood thereby limiting efforts to improve policies and support systems.

Work in emergency departments (ED) is often hectic and unpredictable. Clinicians working in the emergency departments are responsible for delivering care in high-stakes and high stress environments. Regular exposure to a broad scope of patient issues (e.g., diseases, injuries, and traumas), in combination with departmental demands that leaves minimal time for decompression or recovery following a difficult case or patient outcome, may increase clinicians’ risk for psychological distress. [9,15] In fact, when compared to other medical specialties, emergency medicine clinicians have historically experienced greater rates of burnout and post-traumatic stress disorder. [4,5]

To better understand the barriers that emergency department (ED) healthcare providers face in accessing mental health services, this study employed a two-phase mixed methods design. Phase 1 aimed to assess the mental health status of ED providers, including the prevalence of depression and self-stigma, and to identify workplace stressors and barriers to seeking care. Building on these findings, Phase 2 used qualitative interviews to explore providers’ personal beliefs and lived experiences related to mental health care, with the goal of contextualizing and deepening the understanding of the quantitative results. Together, these phases provide a comprehensive view of the mental health challenges faced by ED clinicians and the systemic and personal factors that influence their help-seeking behaviors.

### Methodology

#### Study design.

This study employed a sequential mixed methods design and was conducted in two phases. Phase 1 included a web-based survey provided to all ED physicians, advanced practice providers (APPs; nurse practitioners and physician assistants) and medical residents within the identified medical center. In phase 2, semi-structured interviews were conducted with participants to explore survey findings in greater detail.

#### Ethics statement.

This study was reviewed and approved by the Institutional Review, under protocol number STUDY00018122. All participants provided informed written consent prior to participation. Participants were assured that their responses would remain anonymous and that they could withdraw from the study at any time without consequence. Detailed information of measures taken in each phase of the study to safeguard participant privacy are included below.

### Phase 1 – Survey

#### Survey design.

The survey used in phase 1 included a mix of previously validated instruments [16,17] and scales developed to capture the intended constructs (e.g., occupational barriers and stressors specific to emergency medicine). The identification of target constructs and associated measurement scales was a multi-step process. First, authors performed an in-depth literature review to become familiar with relevant prior research and identify target constructs as well as measurement tools that could be used. For domains lacking validated tools, we constructed items based on published frameworks and expert input. Next, we engaged emergency department providers from a separate institution in a pilot phase to evaluate item clarity, relevance, and comprehensiveness. This included expert review for content validity and cognitive interviews, during which participants verbalized their thought processes while responding to items to confirm appropriate interpretation. Finally, revisions to survey design and wording were made following pilot testing feedback. Revised items were re-tested with a smaller sample of the subject matter experts. This iterative process allowed us to refine the scale, ensuring that the items were both conceptually valid and easily understood by the target population. Although these steps support content validity, formal psychometric evaluation (e.g., internal consistency reliability, factor analysis, convergent/discriminant validity) was not conducted. As such, findings derived from these measures should be considered exploratory. Future research could examine reliability and validity of these measures in larger, more diverse samples to establish their psychometric robustness.

#### Recruitment and sampling.

This study was conducted among emergency department providers within a single academic medical center. The total eligible population included 111 clinicians (physicians, advanced practice providers, and residents). A convenience sample of 43 participants (39% of the total ED staff) completed the survey. Participants were recruited via departmental listserv emails sent weekly over a 12-week period and flyers posted in common areas within the department. Emails included a brief description of the research and a link to the online survey, and flyers included identical language and a QR code for mobile access.

Upon accessing the email link or QR code, participants were redirected to an online eligibility screening survey. Eligibility criteria, identical across both phases of the study, required participants to 1) be a physician, physician assistant, nurse practitioner, medical resident, or fellow within the specified emergency department, 2) willing to participate, 3) proficient in English, both written and verbal, and 4) a United States resident (for compensation purposes). Subjects were ineligible to participate if they were 1) unwilling to participate, 2) unwilling to consent, 3) not proficient in English, both written and verbal, and 4) were not employed as a physician, physician assistant, nurse practitioner, medical resident, or fellow within the specified emergency department. Upon completion of the survey. Those who met all criteria for eligibility were redirected to the informed consent document. Following their review of the document, participants provided implied consent by checking a box labelled “agree to participate” before being redirected to the study survey. Participants received a $20 e-gift card upon completion of the survey. The phase 1 recruitment period opened on June 13, 2022, and closed on September 2, 2022.

#### Data management procedures.

Survey data were collected and managed using REDCap electronic data capture tools [18,19]. REDCap (Research Electronic Data Capture) is a secure, web-based software platform designed to support data capture for research studies, providing 1) an intuitive interface for validated data capture; 2) audit trails for tracking data manipulation and export procedures; 3) automated export procedures for seamless data downloads to common statistical packages; and 4) procedures for data integration and interoperability with external sources. Phase 1 was conducted over a 3-month period. Participants did not provide any common identifiers, as recognized by HIPPA, during the Phase 1 survey. Once the survey was complete, participants were redirected to a second survey, at which point they were asked to provide a name and email address to receive their compensation. Participant data was retrieved from REDCap and stored on a password protected OneDrive account. Only researchers approved during the IRB had access to REDCap survey data.

### Measures

#### Demographic measures.

Demographic data to help characterize the sample was collected during both phase 1 and phase 2. This included age, gender, race/ ethnicity, and marital status. Occupation relevant demographics collected included type of health care professional, time since completion of primary clinical trainings, and years working in ED.

#### Patient Health Questionnaire-9 (PHQ-9).

Providers’ current experiences with depressive symptoms was measured using the Patient Health Questionnaire-9. [17] The PHQ-9 is a 9-item self-report measure to assess depressive symptoms and uses a 4-point response scale ranging from 0 (not at all) to 3 (nearly every day). Items are summed, allowing for interpretation of depression severity: Total scores between 1–4 indicate none-minimal, 5–9 indicate mild depression, 10–14 indicate moderate depression, 15–19 indicate moderately severe depression, and scores between 20–27 indicate severe depression. The measure had good internal consistency (Cronbach’s alpha = .86) in the current study. For ease of interpretation, the PHQ-9 scores were collapsed into 3 broad categories: no to minimal depression (scores of 0 to 4), mild depression (5 to 9), and moderate to severe depression (scores of 10 to 27). In accordance with the procedures mandated by the managing IRB, respondents who indicated an elevated score to item 9 on the PHQ_9 (thoughts of suicide or self-harm), were presented with resources at survey completion, in accordance with the procedures of the governing IRB.

#### Self-Stigma of Seeking Help (SSOSH).

Beliefs and perceptions surrounding seeking mental health treatment were assessed using the Self-Stigma of Seeking Help scale. [16] The SSOSH is a 10-item measure of self-stigma pertaining to seeking psychological help. The scale uses a 5-point Likert scale ranging from 1 (strongly disagree) to 5 (strongly agree). Items are summed and total scores are divided by 10, with possible scores ranging from 1 to 5, in which higher scores indicate greater concern with seeking help from a mental health professional negatively affecting oneself perception. The measure had good internal consistency (Cronbach’s alpha = .81) in the current study.

Barriers to Seeking Mental Health Care – Healthcare Providers List (Barriers to Care)

The Barriers to Seeking Mental Health Care – Healthcare Providers List was created for this study to better understand specific occupational barriers to seeking mental health care that exist among healthcare providers. Participants were asked to indicate if a listed barrier has previously, or was anticipated to in the future, prevent them from seeking mental health care. Barriers included in the list included responses from peers and colleagues, losing face, workplace stigma, fear of paying higher premium for malpractice insurance, fear of losing hospital privileges, lack of available resources, difficulty being a patient, lack of time, and concerns about confidentiality. Participants were also given the opportunity to specify barriers not included in the list in a free text response question. If a potential barrier was selected, this prompted a follow-up question that asked about the participants degree of concern about the selected barrier, using a five-point Likert scale devised for this study ranging from 1 (not at all concerned) to 5 (extremely concerned).

#### Occupational stressors and experiences list (Stressors and Experiences).

The Occupational Stressors and Experiences List was created for this study to capture provider reports of experiences with adverse events experienced by healthcare providers in the workplace. The list was populated by stressors and adverse experiences found to be prominent in literature surrounding workplace stress in emergency departments. Participants were asked to indicate which events they had experienced within the past year (yes/no response). Experiences were grouped into four themes, 1) workload and resource issues, 2) patient and family interactions, 3) workplace safety and environment, and 4) emotional and professional challenges. Examples of experiences asked about included being injured on the job, work overload, insufficient number of staff, overload of administrative work, and negative attitude of profession from patient.

Both the Barriers to Seeking Mental Health Care – Healthcare Providers List and Occupational Stressors and Experiences List were developed through an iterative process informed by literature review, expert consultation, and pilot testing with emergency department providers at a separate institution. Cognitive interviews were conducted during pilot testing to ensure clarity and relevance of items. Although these steps support content validity, formal psychometric evaluation (e.g., reliability, construct validity) was not performed.

#### Evaluation.

General descriptive statistics (means, SDs or frequencies) were reported for demographic (e.g., age, gender, race/ ethnicity, and marital status) and occupation specific characteristics (e.g., type of health care professional, time since completion of primary clinical trainings, years working in ED) to characterize the study sample, both in its entirety and based on clinician type.

For the PHQ-9 and SSOSH scores, descriptive statistics (mean and SD) were generated to provide an overview of the sample’s mental health status. Next, to examine potential differences between groups, independent samples t-tests (using a significance level of 0.05) were conducted to compare mean scores between males and females, as well as between different clinician types. For analysis of responses to the Barriers to Seeking Mental Health Care – Healthcare Providers List and the Occupational Stressors and Experiences List, descriptive statistics were computed to summarize the response frequency among the sample both in its entirety and based on clinician type.

Finally, to explore potential associations between psychological distress and perceived barriers to care, Pearson correlation coefficients were computed between PHQ-9 total scores, SSOSH total scores, and selected items from the Barriers to Seeking Mental Health Care – Healthcare Providers List. These analyses aimed to identify relationships between mental health outcomes and specific barriers reported by participants. SAS software was used for quantitative data analyses (SAS Institute Inc., 2008, Cary, NC, USA).

#### Missing data.

Gender data were missing for one participant. This case was retained in analyses where gender was not a variable of interest and excluded listwise from models incorporating gender. The PHQ-9 was used to assess depressive symptoms. Following standard scoring procedures, if one or two items were missing from a participant’s PHQ-9 responses, the missing values were replaced with the mean of the completed items for that individual. Two observations were missing responses to single items.

### Phase 2 – Interview

#### Epistemological stance and researcher reflexivity statement.

Using an interpretive phenomenological research design (IRP), semi-structured interviews were used in the current study to understand and interpret the experiences of emergency department providers surrounding mental health and accessing mental health resources and make sense of their lived experiences, while acknowledging how these experiences may be influenced by their multiple or intersecting identities. [20] IRP was used to guide the current study, as phenomenology seeks “to describe the essence of a phenomenon by exploring it from the perspective of those who have experienced it”. ([21], pp 670)

As a central component of interpretive phenomenology, the practice of reflexivity attempted to ensure our research team recognized our perspectives and the influence our experiences may have had on interpretation of the data. Our research team was comprised of six members and was conducted at an R1 (i.e., doctoral university with very high research activity per the Carnegie Classification of Institution of Higher Education) land-grant institution in the United States. Interviews were completed by the first author: a female Ph.D. student in behavioral medicine with a background in health psychology and health promotion. The coding team consisted of three female undergraduate students (one freshman, one sophomore, and one senior) who worked as research staff in the same research laboratory. Additional members of the research team include a male internal medicine physician and a male senior research faculty member. We recognize that, despite our efforts, our perspectives likely influenced the study’s direction and outcomes. By maintaining an open dialogue and continuously reflecting on our assumptions and biases, we aimed to minimize the impact of these factors and ensure that the findings were grounded in participants lived experiences.

#### Participants.

The semi-structured interviews were conducted with physicians, advanced practice providers (APPs; nurse practitioners and physician assistants) and medical residents working in the same emergency department as providers in phase 1. Eligibility criteria for phase 2 was identical, therefore participants needed to 1) be a physician, physician assistant, nurse practitioner, medical resident, or fellow within the specified emergency department, 2) willing to participate, 3) proficient in English, both written and verbal, and 4) a United States resident. All study participants provided informed written consent and were given the opportunity to ask any questions prior to the start of interviews. 14/16 interview participants completed the phase 1 survey.

#### Study procedure.

The initial interview guide was developed in conjunction with the phase 1 survey to ensure the interviews would promote further exploration of survey constructs. The first author completed three pilot interviews to ensure interview questions were comprehensible, interview probes were appropriate, and that the interview fostered discussion. Furthermore, the interviewer worked to ensure they were comfortable with the interview guide, with the goal of that comfort translating to greater rapport with participants. Interviews were designed to be semi-structured and conversational by nature, thereby allowing for greater exploration of experiences and ideas. The first author, responsible for conducting all interviews, has extensive training on qualitative research methodologies, with a particular focus on interview techniques, and has conducted research with samples similar to those used in the current study.

Following the completion of phase 1, survey data were analyzed to collect general descriptive statistics to help inform slight modifications to the interview guide. For example, questions were framed to directly reflect survey findings. Example questions include:

- Based on findings from our initial survey, physicians reported feeling moderately supported by hospital leadership, can you tell me a bit more about why you think that might be?
- Do you think there is a stigma surrounding seeking mental health care among providers? [following up on survey measure assessing self-stigma].

Interview questions were purposefully broad to allow for an open dialogue between interviewer and participant, in which the interviewer could follow up with relevant probes to better understand participant experiences. More specifically, the semi-structured interview protocol was designed to explore the mental health resources available to healthcare providers working in emergency departments, the barriers they face in seeking help, and the cultural attitudes surrounding mental health in this high-stress field. The interview began with a reminder of privacy policies and provided an overview of the study’s purpose. Questions focused on personal experiences with mental health resources, coping mechanisms, support systems, and the perceived stigma of seeking help. Additionally, the protocol explored the support provided by leadership, supervisors, and peers. The interview concluded by allowing participants to share any additional insights.

#### Recruitment and sampling.

An email including a brief description of the research and a link to a scheduling survey was sent to subjects who participated in phase 1 and to the department listserv used in phase 1. Subjects who accessed the link provided in the recruitment email were directed to a REDCap survey. Upon accessing the link, subjects were presented with the identical eligibility screening survey used in visit 1. Those who met all criteria for eligibility were redirected to the informed consent document. Participants provided implied consent by checking a box labelled “agree to participate” before being redirected to the study survey. Upon consenting for the qualitative interview, participants registered for a scheduled interview time. All interviews were conducted and recorded using Zoom Video Conferencing software. Although in-person interviews are often the gold standard for qualitative research, conducting interviews via Zoom provided a cost-effective alternative that provided enhanced security and data management features. [22] In addition, it allowed participants to complete the interview in their preferred location (e.g., outside of work) and therefore assisted with their willingness to be more forthcoming. Interview audio files were transcribed using Trint and verified by members of the research team. Participants received a $40 e-gift card for participating in the interview. Phase 2 recruitment period opened on September 19, 2022, and closed on February 2, 2023.

#### Data management procedures.

Upon completion of the screening survey, and deemed eligible, participants were assigned a study ID number that was used to label all study documents associated with them. A single list was maintained that included participants real first initial and full last name, pseudonym, and email. This list was used to store information regarding participant interview details and to avoid confusion through the data collection period. The file containing this list was password protected, and only accessible by the lead researcher. Interviews were strictly audio recorded. Audio files were labeled with participant ID numbers and the date of the interview, before being uploaded to the research OneDrive folder. Information shared during interviews was not shared with employers in any way.

#### Data analysis.

This research utilized the six phases of reflexive thematic analysis identified by Braun and Clarke. [23] Steps include (a) familiarization, (b) generation of initial codes, (c) searching for themes, (d) reviewing themes, (e) defining and naming themes, and (f) producing the report. First, the research team familiarized themselves with the data by listening to the audio, reading the transcriptions and completing any necessary revisions to ensure transcripts were verbatim, and writing memos for each interview. The first author completed a memo immediately following each interview, whereas other team members wrote memos when familiarizing themselves with the interviews. Writing memos allowed for each research team member to make notes about initial impressions and seek clarification about the coding process.

Second, the research team engaged in semantic initial coding process using an open coding approach and electing not to use pre-set codes. In semantic coding, codes capture surface level data and focus on explicit meaning. [24] Each interview was individually coded by two members of the research team, the first or second author and a research assistant, to ensure that different perspectives were considered in the data interpretation process. All codes were reviewed by the first and second author. Third, members of the research team worked independently and collaboratively to search for themes by grouping codes that shared similar meanings. Fourthly, identified themes were reviewed during team meetings at which point the team worked collaboratively to collapse themes that shared the same meaning. Finally, coding disagreements were resolved through consensus and proceeded with naming and defining salient themes. To address potential concerns regarding coding expertise, all interviews were double coded by at least the senior researcher or lead research assistant. Undergraduate coders received structured training in qualitative methods and were closely supervised throughout the process. Coding disagreements were resolved through consensus during regular team meetings, and all codes were reviewed by the first and second authors to ensure consistency. These procedures, combined with researcher triangulation and peer debriefing, were implemented to strengthen the rigor and reliability of the qualitative analysis. MAXQDA software was used for all qualitative data analyses.

#### Trustworthiness and triangulation.

Methods for establishing trustworthiness and triangulation were employed during the five phases described previously, informed by Braun and Clarke. [23] Researchers sought to cultivate trustworthiness by listening and reviewing transcribed interviews, using the practice of writing memos to capture reflexive thoughts that developed during immersion with the data. [25] Data triangulation was achieved by interviewing different provider types who held different identities, educational backgrounds, and diverse professional experiences as healthcare providers. [25] Multiple methods were used to establish researcher triangulation, including the use of diagrams to make sense of theme connections, regular meetings to encourage peer debriefing and assisting members of the research team process their ideas as they evolved during the generation of initial codes, and finally, continued team meetings to establish consensus once themes were established. [25]

## Results

### Phase 1 – Survey

A total of 43 of 111 ED clinicians responded to the survey, yielding a response rate of 39%. The sample included 20 physicians (M age = 41.70), 10 APP’s (M age = 30.90), and 13 residents (M age = 29.38). Complete sample data is presented in Table 1. Among respondents who had completed primary training (not currently in residency), 60% had completed their primary training within the last 5 years.

**Table 1:** Phase 1 Sample Demographics.

	Total (N = 43)	Physicians (N = 20)	Advanced Practice Providers (N = 10)	Residents (N = 13)
Gender [N (%)]				
Cisgender female/ woman	20 (47.62)	8 (40.00)	7 (70.00)	5 (41.67)
Cisgender male/ man	22 (52.38)	12 (60.00)	3 (30.00)	7 (58.33)
Unidentified				1 (7.70)
Age [Mean ± SD]	35.6 ± 10.43	41.70 ± 11.33	30.90 ± 7.75	29.38 ± 2.81
Marital Status [N (%)]				
Single	15 (3.88)	5 (25.00)	3 (30.00)	7 (53.85)
Married or in a domestic partnership	28 (65.12)	15 (75.00)	7 (70.00)	6 (46.15)
Race [N (%)]				
Asian or Pacific Islander	8 (18.60)	5 (25.00)	–	3 (23.08)
Black or African American	1 (2.33)	1 (5.00)	–	–
Hispanic or Latino	4 (9.30)	2 (10.00)	–	2 (15.38)
White or Caucasian	29 (67.44)	12 (60.00)	9 (90.00)	8 (61.54)
A race/ethnicity not listed here	1 (2.33)	–	1 (10.00)	–

### Survey findings

#### Patient health questionnaire-9.

The mean score on the PHQ-9 was 6.09 (indicative of likely mild depression). Overall, 11 (25.59%) respondents scored in the moderate to severe depression range on the PHQ-9, 7 (63.6%) of whom were women. Conversely, 71.43% of men presented with none to minimal depression. When scores for depression were compared for men and women represented within this sample, women (M = 8.05; mild depression) scored higher than their male colleagues (M = 4.70; none-minimal depression), a difference sound to be statistically significant (t(38) = 2.06, p = 0.05), with a medium to large effect size (Cohen’s d = 0.65, 95% CI [0.06, 6.72]). When stratified by clinician type and gender, female APPs had the highest mean PHQ-9 score of 9.14 (mild depression), followed by female residents (M = 8.4; mild depression), and finally male residents (M = 6.71; none-minimal depression).

#### Self-stigma.

The mean score of self-stigma, scored on a scale of 1–5, among the complete sample was 2.51 (SD = 0.60), higher than previously found (M = 1.64) among a sample of primary care providers (PCPs) from the United States [26]. When scores for self-stigma were compared for men and women represented within this sample, men had higher scores (M = 2.81, SD = 0.54) than their female colleagues (M = 2.20, SD = 0.52), a difference found to be statistically significant (t(40) = -3.71, p = 0.001), with a large effect size (Cohen’s d = 1.15, 95% CI [0.28, 0.94]). When stratified by clinician type, the mean score was highest among physicians (M = 2.6), followed closely by residents (M = 2.52), and finally APPs (M = 2.28). When stratified by both gender and clinician type, self-stigma remained highest among male providers, specifically male physicians (M = 2.78).

#### Barriers to seeking mental health care – healthcare providers list.

‘Lack of time’ was the most cited barrier across all providers; however, provider groups varied significantly beyond this commonality. Barriers reported most frequently by physicians included ‘invasion of privacy’ (60%) and ‘concerned about confidentiality’ (45%). Barriers reported most frequently by APPs were ‘lack of available resources’ (50%) and “belief treatment will not help’ (50%). Finally, barriers most reported by residents included ‘medical board licensing protocol’ (62%), ‘workplace stigma’ (46%), and ‘invasion of privacy’ (46%). For all barriers identified by provider groups, participants indicated only being ‘somewhat concerned’. A complete list of barriers to seeking mental health care among the whole sample, and top endorsed barriers by provider type, can be found in Table 2.

**Table 2:** Barriers to seeking mental health care.

Barriers	% Endorsing (n)	Degree of Concern	SD
**Structural Barriers**			
Lack of time	72.09 (31)	4.35	0.88
Medical board licensing protocol	37.21 (16)	3.88	1.09
Fear of paying a higher premium for malpractice insurance	27.91 (12)	3.83	0.94
Lack of available resources	25.58 (11)	3.27	0.9
Fear of workplace disciplinary actions	18.60 (8)	3.75	1.28
Fear of losing hospital privileges	16.28 (7)	3.71	1.38
**Attitudinal Barriers**			
Invasion of privacy	48.84 (21)	3.38	1.07
Belief that treatment will not help	37.21 (16)	3.5	0.63
Concerned about confidentiality	34.88 (15)	3.67	0.98
Workplace stigma	32.56 (14)	3.21	0.97
Difficulty being a patient	32.56 (14)	3.57	0.76
Personal need to portray good health	30.23 (13)	3.54	0.78
Response from peers and colleagues	25.58 (11)	3	0.89
Losing face	18.60 (8)	3.5	1.07
Perception of weakness by peers/ colleagues	18.60 (8)	3.88	0.83
**Most Endorsed Barriers by Provider Type**			
**Barriers**	**% Endorsing (n)**	**Degree of Concern**	**SD**
**Physicians**			
Lack of time	70.00 (12)	4.50	0.94
Invasion of privacy	60.00 (12)	3.42	1.08
Concerned about confidentiality	45.00 (9)	3.56	1.13
**APPs**			
Lack of time	90.00 (9)	4.11	0.93
Lack of available resources	50.00 (5)	3.60	0.55
Belief that treatment will not help	50.00 (5)	3.60	0.55
**Residents**			
Lack of time	84.62 (11)	4.36	0.81
Medical board licensing protocol	61.54 (8)	3.38	1.19
Workplace stigma	46.15 (6)	3.33	1.03
Invasion of privacy	46.15 (6)	3.67	1.03
Fear of paying a higher premium for malpractice insurance	46.15 (6)	3.83	1.17

#### Occupational stressors and experiences.

‘Unrealistic expectations of patients and family members’ was the most cited experience across the complete sample, however experiences among provider groups varied beyond this commonality. Within individual provider groups, ‘insufficient number of staff’ was a prominent experience, and most endorsed by residents (92.31%). A complete list of occupation specific experiences encountered by providers within the whole sample, and top endorsed experiences by provider type, can be found in Table 3.

**Table 3:** Occupational Stressors and Experiences.

Experiences	% Endorsing (n)
**Structural Stressors**	
Insufficient number of staff	88.37 (38)
Work overload	72.09 (31)
Daily unforeseen and unplanned-for situations	62.79 (27)
Limited time to examine patients	60.47 (26)
Overload of administrative work	58.14 (25)
Lack of resources to perform occupational duties	55.81 (24)
Injured on the job	9.30 (4)
Physical altercation with patient or patient family	9.30 (4)
**Attitudinal Stressors**	
Unrealistic expectations of patients and family members	90.70 (39)
Negative attitude of profession from patient	65.12 (28)
Compassion fatigue	62.79 (27)
Verbal harassment from patient or patient family	48.84 (21)
Hostile work environment	34.88 (15)
Physically unsafe working conditions	16.28 (7)
**Most Endorsed Experiences by Provider Type**	
**Experiences**	**% Endorsing (n)**
**Physicians**	
Unrealistic expectations of patients and family members	90.00 (18)
Insufficient number of staff	85.00 (85)
Work overload	65.00 (65)
**APPs**	
Unrealistic expectations of patients and family members	100.00 (10)
Insufficient number of staff	90.00 (9)
Daily unforeseen and unplanned-for situations	90.00 (9)
**Residents**	
Insufficient number of staff	92.31 (12)
Unrealistic expectations of patients and family members	84.62 (11)
Work overload	76.92 (10)
Limited time to examine patients	76.92 (10)

#### Exploratory analyses.

There was a positive correlation between PHQ-9 total scores and workplace stigma (r [41] = 0.42, p = 0.006). Positive correlations were also observed between SSOSH total scores and lack of time (r [41] = 0.32, p = 0.04), invasion of privacy (r [41] = 0.43, p = 0.004), and belief that treatment would not help (r [41] = 0.35, p = 0.02).

### Phase 2 – Interviews

#### Sample characteristics.

A total of 16 ED clinicians participated in an interview, 14 of whom completed the phase 1 survey. The sample included 6 physicians (M age = 48.5), 8 APPs (M age = 34.67), and 2 residents (M age = 27.5). Participant demographics are available in Table 4. The provider population of the emergency department sampled is predominantly White and therefore race and ethnicity for phase 2 participants is not included to protect participant identity. To further protect the privacy and confidentiality of participants, pseudonyms are used throughout this report, as seen in Table 5.

**Table 4:** Phase 2 Sample Demographics.

	Total (N = 16)	Physicians (N = 6)	Advanced Practice Providers (N = 8)	Residents (N = 2)
Gender [N (%)]				
Cisgender female/ woman	9 (56.25)	4 (66.67)	4 (50.00)	1 (50.00)
Cisgender male/ man	7 (43.75)	2 (33.33)	4 (50.00)	1 (50.00)
Age [Mean ± SD]	38.29 ± 12.23	48.5 ± 8.55	31.67 ± 9.56	27.5 ± 0.71
Marital Status [N (%)]				
Single	6 (37.5)		5 (62.5)	1 (50.00)
Married or in a domestic partnership	10 (62.5)	6 (100.0)	3 (37.5)	1 (50.00)
Race [N (%)]				
Non-White	4 (25.00)	4 (66.67)	–	–
White or Caucasian	12 (75.00)	2 (33.33)	8 (100.0)	2 (100.0)

**Table 5:** Interview Participant Information.

	Name*	Clinician Type	Gender	Age	Years Since Primary Clinical Training	Years in ED
1001	Claire	Physician Assistant	Female	25	<5 years	1-3 years
1002	Anthony	Physician	Male	39	6-10 years	10 + years
1003	Melissa	Physician	Female	53	21 + years	10 + years
1004	Stephanie	Physician	Female	37	6-10 years	7-9 years
1005	Heather	Resident	Female	28	In-Training	PGY-1
1006	Kelly	Physician	Female	50	21 + years	10 + years
1007	Michelle	Physician Assistant	Female	49	16-20 years	10 + years
1008	Maria	Physician	Female	58	21 + years	10 + years
1009	Dustin	Resident	Male	27	In-Training	PGY-1
1010	Jamie	Physician Assistant	Male	26	<5 years	1-3 years
1011	Rachel	Physician Assistant	Female	–	16-20 years	10 + years
1012	Jeremy	Physician Assistant	Male	–	<5 years	1-3 years
1013	Logan	Physician Assistant	Female	24	In-Training	1-3 years
1014	Aaron	Nurse Practitioner	Male	36	6-10 years	6-10 years
1015	Thomas	Physician Assistant	Male	30	In-Training	10 + years
1016	Donald	Physician	Male	54	21 + years	10 + years

*Pseudonym.

#### Interview findings.

**Fig 1:**
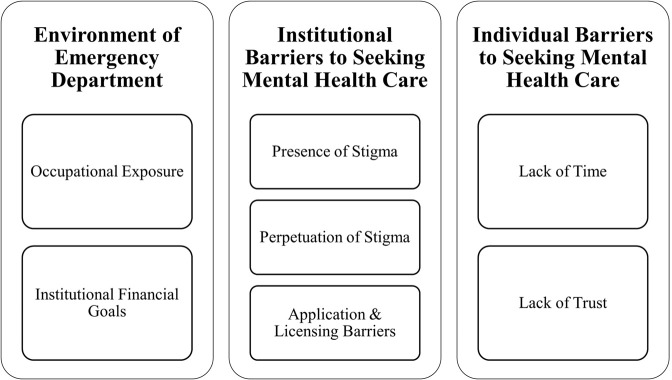
Major themes and sub-themes.

Participants discussed their perceptions of the status of mental health among ED providers, stigma surrounding providers seeking mental health care, the need for, and availability of, mental health resources for providers, and experiences unique to working in the emergency department that may negatively affect provider well-being. When participants discussed their personal experiences as ED providers, the following three major themes (Fig 1) were identified: 1) environment of emergency department, 2) mental health related stigma, and 3) barriers to seeking mental health care. Thematic coding revealed additional subthemes; in the following section, subthemes are organized under major themes.

All participants interviewed endorsed the belief that there is an undeniable need for mental health resources designed specifically for providers within this ED. Providers interviewed shared that the high stress of working in the ED made the need for mental health resources a prominent issue. When asked what contributed most to the high stress environment, participants most notably discussed the culture of emergency medicine, competition among providers, and institutional goals for revenue generation.

#### Occupational exposure.

Emergency medicine is widely recognized as a high-stress environment due to several factors, including urgency and acuity of cases, unpredictability, long hours, and resource constraints. As such, maladaptive coping mechanisms, such as avoidance or emotional numbing, are commonly endorsed and culturally accepted norms within EM, and explored further in theme 2. Therefore, it is understood why participants unanimously concurred that it is imperative for resources to be available for providers within the ED. Claire, a physician assistant who at the time of the interview had recently started in the ED, discussed the need for resources to address high rates of burnout among providers and how instead of seeking regular care, many providers only reach out during times of crisis. She said,

I think the need is super high. Our profession, particularly the specialty of emergency medicine, is very emotionally and mentally taxing and we talk about burnout all the time, like it’s a topic that comes up nearly constantly. And I think that burnout rates and people feeling burned out is so prevalent because of not seeking mental health care at a baseline and people only going when things are crisis level (1001, physician assistant).

Further evidence of the need for resources in the ED was provided by Jamie, a physician assistant. He emphasized the necessity of resources due to the fast-paced and high-pressure environment and highlighted the additional need because of the frequent exposure to difficult and potentially traumatic cases provider’s encounter. He shared,

I think definitely there needs to be an availability across the board and hopefully better advertised as well…and I think it’s definitely good to have resources in place to be able to talk about that and get past some of these different patients because, I mean, you’re working multiple days in a row, you have multiple exposures to these and it can be difficult and I think an outlet would be very valuable as well (1010, physician assistant).

Finally, a sentiment emerged that the ED environment differs from that of other medical specialties. One key difference is the demands working in the ED places on providers. Melissa, a senior physician, articulated how demanding working in an emergency department can be, specifically at an academic medical center, and the stress induced by all the demands taking place simultaneously. She shared,

There’s so many things that are imposed upon you. You need there’s the patient volume, there’s, you know, getting the diagnosis right. There’s also the rapport with the patient. There’s so many factors, the RVUs, you have to like it’s the money situation, too, in terms of you need to see more patients...you need to do the scholarship. You need to do the research. You need to teach the residents. You need to be good at procedures. You need to be good diagnostically. You need to be good at teaching. So there are so many things that are put upon you that you need to be good at and it’s expected to you, not to mention. And that’s outside of just being in the space. You mean you have to actually you have to adapt pretty quickly because so many things are thrown at you. You don’t know what’s going to happen when you walk through the door (1003, physician)

#### Institutional financial goals.

The general theme that emerged from the interview participants was the understanding that the hospital, just as any other business, functions to make a profit. Participants shared their belief that for the institution to make that profit, they were required to see more patients; this, in turn, amplified stress among providers and was thought to negatively affect provider well-being. The feeling of pressure to perform for providers in this emergency department was magnified as they felt constantly bombarded with institutional metrics demonstrating performance, as pointed out by Rachel, a senior PA. She shared,

I feel like we, we are all under the same this like pressure to perform and make sure to meet so many metrics you know we have so many metrics you know we get them thrown in our faces every week you know door to doc times, door to disposition times, you know how many patients are seeing per hour and you know are your charts being filled out completely (1011, physician assistant)

Although those metrics align with the institutional goal to be profitable, the perception among a majority of participants was that the institution striving to meet this goal has had a detrimental effect on the well-being of providers. Anthony, a physician who had worked in emergency medicine for 10 + years, shared that he felt as though his well-being was not a concern for the hospital unless it affected the bottom line and that he was dispensable and could be replaced. He said,

I would say fact that they don’t care about my well-being...they’re actually very overt about it, which is kind of nice. I know. I know what I’m getting into…but yes it’s not it’s not for my well-being, anything that’s being done. It’s for the purpose of the bottom line. And the bottom line isn’t my psychiatric betterment, unless it helps their bottom line, which in theory it should. But it’s easier to just hire somebody else (1002, physician).

More experienced participants noted that the provider-patient relationship has evolved due to the increasing emphasis on revenue generation in emergency medicine. Donald, a 20 + year veteran ED physician, discussed his frustrations about patient care decisions now being made by administrative criteria, determined largely by insurance companies willing to cover patient expenses. He shared the added stress for providers that comes with being unable to care for patients in the way they want. He said,

it’s [money] the driver for everything. Yeah. It’s all about dollars. If I. If I am um. Motivated as an administrator by that amount of money um to where to do good things. And I’m not saying that you shouldn’t make money. I’m not saying that you shouldn’t be profitable because I think you should um um but um it sometimes oversteps sort of the interpersonal thing, like, what’s the right thing to do? Like this person with a brain abscess who’s sitting in a chair in the emergency department, probably still sitting there, you know, five days later um. Boy, it seems wrong, right? that just seems wrong. Right. But that’s not what this. That’s. That’s not the narrative. The narrative is they don’t meet criteria by some administrative thing that’s mostly insurance driven and payment for hospitalization driven. And so, therefore, we have to find alternatives of care, but yet we don’t have the infrastructure or actual alternatives for that care so the poor wife and daughter who are working so hard to take care of dad just can’t do it anymore. They’re at wits end, too (1016, physician).

Participant interviews identified how several factors were thought to contribute to ED providers experiencing a high level of stress. Participants shared both the emotional and physical toll experienced working in the ED and how they feel that contributes to providers experiencing burnout. Finally, they report that the shift in institutional priorities from patient-centered care to revenue generation forces providers to make difficult decisions about patient care. These factors, among others, result in a work environment in which providers feel as though their well-being is not fully supported or valued.

### Theme 2: Institutional barriers to seeking mental health care

#### Presence of stigma.

Each participant discussed their perceptions about the presence of stigma within emergency medicine, including how the presence of stigma impacts how individuals are perceived by their peers if they were to seek mental health care. Maria, a female physician with over 20 + years of experience in the ED, highlighted the presence of stigma related to mental health care. She pointed out that individuals who seek and receive mental health support may face negative labeling from their peers. She shared,

There’s a stigma attached to having needed or worked with mental health professionals. Other people will say they don’t have the time. They’re too busy. I think people don’t want to be labeled in a certain way, so they wouldn’t want others to know that they were maybe not strong enough or don’t have the capabilities to deal with the type of work they have or they do (1008, physician).

Heather, a female intern who at the time of the interview had only been working in the ED for a couple months, also discussed the presence of stigma surrounding negative peer perception if someone is labelled as ‘weak’. When asked about how stigma may serve as a barrier to seeking mental health care, she said,

you don’t want to show that you’re struggling because if you do, it looks like you’re weak or you weren’t made for this job, especially, I think as a whole, and especially E.R. in like more high-pressure environments or specialties. But you definitely don’t want to show like your weaknesses because you feel like you’re falling behind or you’re not progressing adequately, and you don’t want anyone to think that you’re not doing a good job (1005, resident).

Furthermore, multiple participants expressed the view that providers in the emergency department are expected to be unaffected by their job regardless of what they encounter at work, such as high acuity patients, traumatic accidents, patient deaths, department overcrowding, etc. Jamie, a male PA who recently started working in the ED, shared his perceptions about mental health stigma and how it is affected by unhealthy expectations of providers continuing to be upheld. When talking about his experience, he said,

But even in my short time, it does feel like there’s a little bit of underlying stigma, whether it’s not overtly voiced, but it seems like it kind of is lying there and dormant and just it’s kind of across the board. It seems like no one no one is supposed to go get mental health. No one is supposed to be upset about a case… It’s not talked about as much about making sure you’re mentally healthy, coming in to work every day. It’s just kind of you’re expected to hit the ground running, you know, and then just crank through your whole shift and then finish out. And then if you see a difficult case along the way, you know maybe talk to a colleague, but I don’t I don’t feel like there’s any specific thing [policy or procedure] (1010, physician assistant).

This represents the majority of responses from participants interviewed in this study.

#### Perpetuation of Stigma.

Beyond current perceptions of stigma, participants routinely discussed how their past and current experiences shaped their behaviors that contribute to the perpetuation of mental health related stigma. Examples of these experiences included the environments they trained in, the respect they wanted to maintain from other providers in the department, and their attitudes about how they will train their subordinates. For instance, Melissa, a senior level physician who had worked in the ED for 20 + years discussed how the environment she trained in, and her time in the military, shaped the way she perceived mental health as a weakness. She shared,

And I’m old school too, so maybe the younger generation. But...I had grown up in a patriarchal school in the late nineties. I went to medical school and there’s a lot of hazing there. But then I also was in the military in the early in the late nineties, early 2000s and then went to residency after that in the south where it’s very much an old boys school down the south. So I have a very so my personality is skewed in that sense, but it’s looked at as weakness. If you can’t like if you don’t have the mental fortitude to kind of pull yourself up by your bootstraps and soldier on. So it’s sort of like, suck it up, Suzy, let’s go (1003, physician).

Melissa went on to discuss how, particularly female physicians, are viewed negatively by nurses and other staff if they were to ‘breakdown’ in front of them. She discussed coaching her female residents in a manner similar to her own training, teaching them to maintain their emotions until they are somewhere private as a survival mechanism to save them from negative peer perceptions and gossip. She shared,

Like to tell the residents, don’t show emotion, don’t show. But you really like honestly, it’s in her best interest not to cry in front of the nurses. You can’t break down because you will be talked about… But I’m the residents, especially the female residents, have always come to me because, you know, I am I’m hard core, but I do care about them and want to make sure that they can survive residency and that their well-being is fine. But I also don’t want them to be labeled as, you know, one that can be easily that’s that’s weak and cries at the drop of a hat or if a nurse yells at them, that they’re going to fall apart into a puddle and like, disappear (1003, physician).

The broad culture within the field of emergency medicine as a whole was also identified as a contributing factor to the perpetuation of mental health related stigma. More specifically, Donald, a 25-year veteran physician familiar with the administrative responsibilities of running an emergency department, described the state of emergency medicine in the United States as ‘a safety net for health care’. Therefore, it is his belief that emergency medicine attracts individuals with certain personality types that self-select in to working in these types of environments, therefore contributing to the perpetuation of mental health related stigma. He said,

So, I think…there are [personality types] generally drawn to that type of, you know, run towards a fire [instead of] away from the fire type mentality um that has historically been, you know more of the hero than it is the the victim. Right. And so, um you know, sometimes people see themselves as invincible and all that other stuff related to it…people that have that general mentality related to it that they, you know, they’re there to help and fix as opposed to, you know, uh be injured themselves and be fixed (1016, physician).

In addition, providers experiences working in the emergency department and the type of patient interactions they encounter was also believed by many to be a factor that negatively contributes to an individual’s self-perception, ultimately having adverse effects on self-stigma and contributing to the continued perpetuation of mental health stigma. When asked what she thinks perpetuates mental health stigma, Maria, a senior physician, shared,

I mean, I think people in medicine are, you know, that’s your job. You deal with this every day like, you know, you got that kind of tough it out. You’ll be fine. This happens to all of us. Don’t worry. And then I think we do see people with really bad mental illness in the emergency department. You don’t want to equate yourself to someone that in your mind is really sick. You know, there’s something wrong with them (1008, physician).

#### Application and licensing barriers.

Efforts have been made by health care organizations and various medical organizations to address barriers to seeking mental health care established by state licensing boards. For example, working to remove regulations that punish clinicians for seeking mental health support or care. Despite such efforts, over half of resident survey participants in the current study indicated ‘medical board licensing protocol’ as a barrier to seeking mental health care. When intern Dustin was asked during his interview about barriers, he discussed that among peers in medical school there was a belief that seeking out mental health care could jeopardize one’s application to residency. He shared,

I think that’s part of the concern during medical school is getting into residency. And I certainly think that people avoid pursuing mental health counseling at school due to concerns over decreasing their competitiveness in their application (1009, resident).

Specifically, as it related to initial or renewal applications for state medical licenses, Rachel a senior PA, discussed the potential implications of disclosing having sought out mental health care services. She said,

Just because it’s asked so many times, it’s asked when you get your state license, it’s asked when you get credentialed. And so, it usually comes along with these other questions of, you know, have you committed any crimes? Have you, you know, been under any legal action? So when they’re asked almost in the same paragraph… You kind of lump it all together and it seems like it’s criminal to have a mental health disorder (1011, physician assistant).

Rachel went on to share the struggle to be honest many may encounter when answering the licensing questionnaires. When asked about the follow-up questions if someone indicates having sought mental health treatment, she said,

[if positive] then you have to have an explanation and sometimes documentation to back it up as to what in what way or, you know. Have you had any addiction problems and how did you handle that? Or have you ever been reprimanded by the board for or reported to the board for having addiction problems or anything of that nature? And so, yeah, I mean, I feel like that can be used against you because if there there’s always that fear if you are sued, what, what, you know, where does it end with the personal space? What do they have access to? I don’t know the answer to that because of not being under a deposition in the past. I don’t know what they can use against you. Yeah (1011, physician assistant).

Participant interviews revealed reports of experiences highlighting a complex interplay of factors shaping the stigma surrounding seeking mental health care in emergency medicine. Participants discussed how seeking mental health care may have negative effects on how individuals are perceived by their peers and therefore feel pressured to appear strong and unaffected by the challenging nature of working in the emergency department. The perpetuation of stigma from older providers within the profession, who may have been influenced by traditional and patriarchal views, generally discouraged showing emotion (let alone help-seeking) for risk of being viewed as weak. The exacerbating effects of fear brought on by the potentially negative implications seeking care could have on their license applications.

### Theme 3: Individual barriers to seeking mental health care

Participants each acknowledged barriers that have previously kept them personally, or their peers, from seeking mental health care. Based on experiences shared by participants, these barriers were broadly identified as reflecting [1] lack of trust and [2] lack of time.

#### Lack of trust.

Lack of trust was seen as a primarily intrapersonal barrier; intrapersonal barriers were operationalized within the current study as barriers that stem from an individual’s attitude, beliefs, behavior, habits, or personality. Experiences shared by participants indicate that perception of barriers is shaped, in part, by their experiences as health care providers within their organizations. Kelly, a senior level physician, shared what she believes are the two main barriers for individuals seeking mental health care,

Well, I think I think those are the two major ones. Like you don’t trust your hospital and you don’t really want to admit that you need to go seek help… I think the biggest barrier is getting someone you can trust. There has been I mean, if you look online, there has been a lot of talk about employee assistance programs that eventually are harmful to the physician and their career (1006, physician).

When asked to expand on her statement about having a lack of trust in the hospital, Kelly spoke directly about the effectiveness of programs and the lack of confidentiality. She said,

I don’t really know how effective the programs are, because honestly, half the time that administration talks about wellness, you don’t really they don’t I don’t think they really understand it from a physician point of view. So I don’t know if any of their programs were actually be effective. And B, again, there’s a confidentiality thing, and I think that is tricky. And then also, you know, you see people who have said, yeah, I, you know, I’m depressed or something and like, you know, three months later, they’re shuffled off to their state program where they’re forced. I’ve heard horror stories about that (1006, physician).

Stephanie, a physician who has personally sought mental health care from providers within the institution shared her perspective about provider trust in the institution, based both on her own experience and discussions with peers. She shared,

There are going to be some people who are suspicious of anything that comes from the hospital in terms of protecting their confidential information. So I’m not one of those people. But I did feel like I did just conversations with my, some of my coworkers (1004, physician).

Unfortunately, the attitude regarding a lack of trust in the institution was expressed by multiple participants and appeared to be deeply rooted in personal fear. Maria, also a senior physician, recognized colleagues who think they are ‘fine’ or ‘do not need help’ may be fearful of finding out the opposite is in fact true. She said,

I think some people will think that they’re fine and they don’t need the help or, you know, another perspective for someone to listen to them. I think some people are afraid (1008, physician).

#### Lack of time.

Recognized among survey participants as the most common barriers, interview participants also discussed ‘lack of time’ as a barrier to accessing mental health resources. As a physician, Stephanie noted all the responsibilities that make finding time to schedule an appointment difficult. She shared,

And I think, you know, when you’re a practicing physician working full time and picking up, you know, extra shifts and then you have your administrative and non-clinical duties, it doesn’t leave much time for you to make appointments or, you know, get to appointments within a reasonable time. And sometimes you’re just so exhausted that, you know, even taking that step is a barrier (1004, physician).

Beyond clinical and occupational responsibilities, many participants identified as spouses, parents, or caregivers and discussed the responsibilities they are trying to balance. As a result, occupational and familial responsibilities were regularly described as taking priority over scheduling personal health appointments, which were seen as time away from family or loved ones. Michelle, a PA, shared,

So I think, you know if you have a family at home, you just want to get home, um, I don’t think there’s you don’t give yourself or there isn’t that time because till you get home, they do what you need to do. Go to bed, get up the next day, maybe the kids are gone or whatever. You just you don’t have that. I don’t think a lot of people really can get a lot of time to actually do it (1007, physician assistant).

Similarly, among residents whose schedules in the ED vary so greatly from week to week, ‘lack of time’ proved to be a significant barrier as well. Dustin, a resident, discussed that because of the resident scheduling procedures it is difficulty to make any type of medical appointment, including mental health care. Furthermore, he discussed the common behavior among trainees to neglect their health. He shared,

It [resident schedule] makes it really difficult to schedule something in advance when oftentimes you don’t know your schedule for three weeks out. Beyond that, most of those places require it. So. It’s very difficult to actually schedule something and feel any kind of confidence at all that you’re going to be able to make it… I think that it’s not uncommon for medical students as well as residents to neglect their health in more ways than just mental, but also physical. It’s really hard to get appointments of any kind of like PCP or vision or dental whenever you work before and after those offices close (1009, resident)

Collectively, interview participants highlighted experiences with the complex interplay between individual, institutional, and regulatory factors contributing to the challenges healthcare providers face in seeking mental health care. The identified barriers, including trust issues, time constraints, and concerns about professional repercussions, shed light on the need for comprehensive support systems to support health care providers interested in seeking mental health care.

### Integration of findings

**Fig 2:**
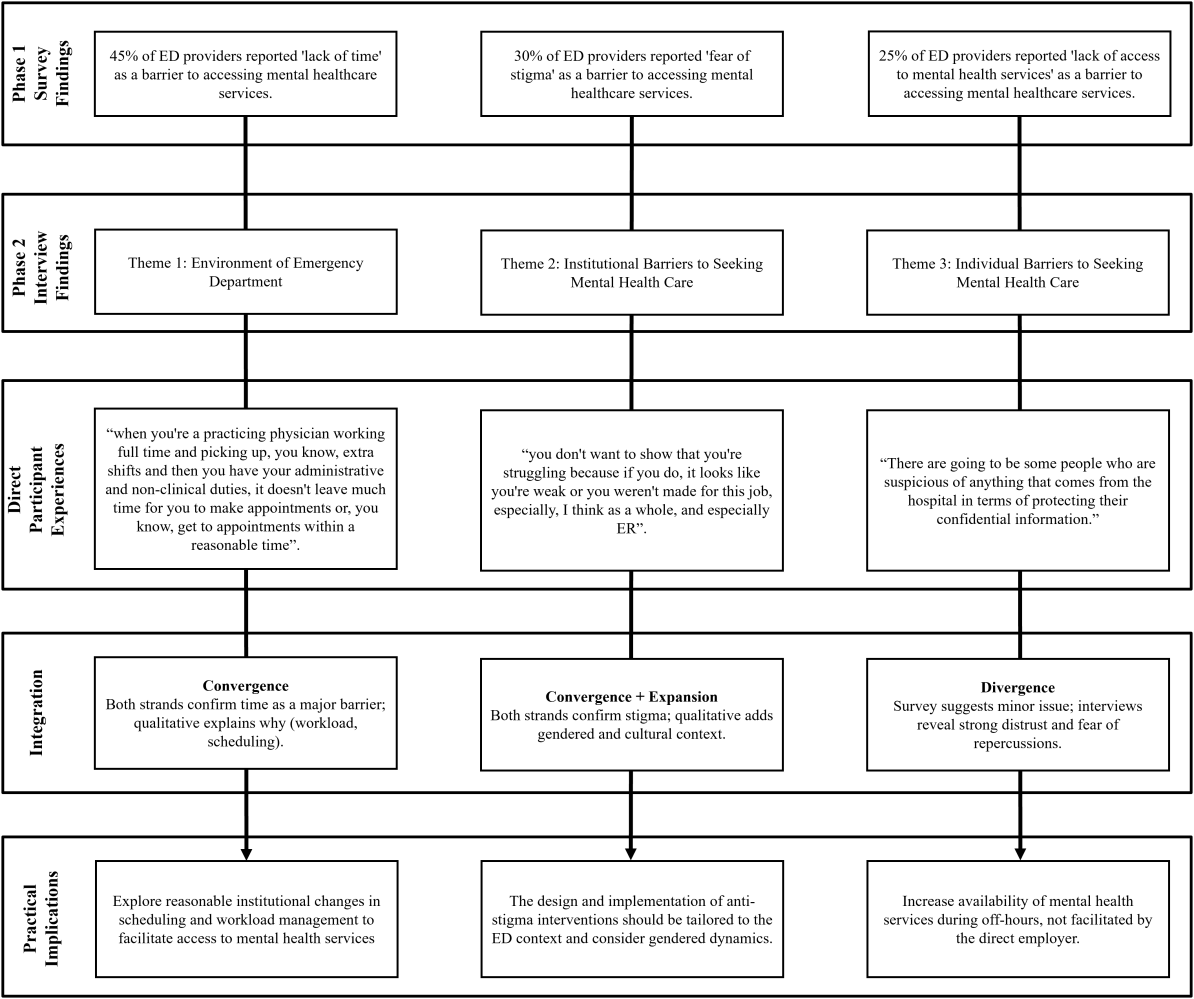
Integration of Findings – joint display.

This section presents the integration of both sources of data to provide a more comprehensive understanding of the research objectives. The integration highlights how the qualitative themes help explain or expand upon the statistical trends identified during phase one. To support this examination, a joint display (Fig 2) was developed to better illustrate areas of convergence and divergence between data sources and to highlight practical implications.

Findings converged significantly when exploring stigma. Survey results indicated moderate levels of self-stigma, particularly among male providers. This was reinforced by interview data, where participants described a culture of emotional suppression and fear of being perceived as weak. A resident shared, “You don’t want to show that you’re struggling because if you do, it looks like you’re weak or you weren’t made for this job...” This convergence supports the need for stigma-reduction efforts tailored to the ED context. A clear convergence was observed regarding time constraints. Quantitatively, 72% of participants identified lack of time as a barrier to seeking mental health care. This was echoed in the qualitative interviews, where participants described the demanding nature of ED work and the difficulty of scheduling appointments. For example, one physician noted, “...it doesn’t leave much time for you to make appointments or, you know, get to appointments within a reasonable time.” These findings suggest that institutional scheduling practices may be a critical target for intervention.

A divergence emerged in perceptions of access and trust. While only a minority of survey respondents endorsed lack of access as a barrier, qualitative data revealed concerns about confidentiality and institutional trust. One physician stated, “There are going to be some people who are suspicious of anything that comes from the hospital in terms of protecting their confidential information.” This suggests that while services may be technically available, they may not be perceived as safe or usable by providers.

Together, these findings underscore the multifaceted nature of barriers to mental health care in emergency medicine and point to actionable areas for institutional change, including workload management, stigma reduction, and trust-building around mental health resources. Finally, these integrated insights set the stage for a broader discussion of their implications for clinician well-being, institutional policy, and future research.

## Discussion

This mixed methods study explored the prevalence of self-reported depression and self-stigma, as well as barriers to seeking mental health care among clinicians working in the emergency department. Through theoretically informed surveys and qualitative interviews, study findings contribute to a growing body of literature helping to extend our understanding of the mental health challenges faced by healthcare professionals. Furthermore, findings highlight factors that may contribute to suboptimal clinician well-being and have potential implications for occupational outcomes such as clinician retention and quality of patient care.

Although psychological distress among health care providers within the United States is high, mood disorders and mental illness often go unnoticed and unreported. Previous findings indicate this contributes to the higher prevalence of burnout and other negative workplace and well-being consequences in comparison to other US workers. [11,12,27] Phase one survey results indicated that nearly one-third of participants reported moderate to severe depressive symptoms, a finding inconsistent with previous work that suggested majority of providers reported ‘no tentative diagnosis of major depression’ (tentative diagnosis of major depression defined as PHQ-9 = > 10). [28] This discrepancy may reflect differences in timing, as the current study was conducted in 2023, a period marked by sustained workforce strain and evolving institutional pressures during the COVID-19 pandemic. These findings align with broader evidence that psychological distress among healthcare workers remains elevated and often underreported. [12,29]

Self-stigma was also found to be moderate, with significantly higher levels among male providers. These observed gender differences in self-stigma may reflect entrenched cultural norms within medicine that valorize stoicism and emotional resilience and discourage emotional vulnerability. [26] This is particularly prominent in high-intensity specialties like emergency medicine, where ”hero culture” may amplify these expectations, fostering a reluctance to seek psychological support. [30] Structural factors, including hierarchical training models and performance metrics, further reinforce these norms by signaling that vulnerability may jeopardize professional credibility. Notably, male physicians exhibited the highest stigma scores, suggesting that professional role may intersect with gender to shape help-seeking attitudes. Previous work suggests male physicians, in particular, tend to suppress emotional expression, leading to reduced help-seeking behavior, poorer mental health outcomes, and increased burnout. [12] For residents, experienced stigma may be compounded due to hierarchical training structures [31] and fears that seeking mental health care could negatively affect evaluations, licensure, and long-term career prospects; concerns documented as significant deterrents to help-seeking among trainees. Although not the primary focus of this study, this gender difference warrants further exploration, particularly in relation to professional identity and help-seeking behavior.

The findings are further contextualized by stigma theory, which posits that perceived social judgment can inhibit individuals from seeking care. [16] This was evident in both the quantitative and qualitative data, where concerns about being perceived as weak or unfit for working as a provider in the emergency department were commonly expressed. A 2024 study by Zamorano [10] found stigma towards mental health conditions was a significant predictor of burnout, specifically emotional exhaustion and depersonalization, among healthcare professionals, reinforcing the importance of addressing stigma as both a cultural and clinical concern.

Within the current study, barriers to seeking mental health care extended beyond stigma. Lack of time was the most frequently endorsed barrier across all clinician groups, echoing findings from Rink [32] whose findings parallel the NAM’s Systems Model of Clinician Burnout distinction between work system factors and individual mediating factors. More specifically, findings indicated that time specific stressors (e.g., lack of time to schedule appointments) were pervasive, especially in relation to workload and scheduling, and contributed to work-life imbalance. [32] While Rink [32] identified time constraints as a barrier, phase two interview findings within the current study added qualitative depth by illustrating how these constraints are experienced in the ED context, reinforcing the NAM model’s emphasis on the interplay between systemic and individual-level factors in clinician burnout. Interview findings further revealed that even when services were technically available, concerns about confidentiality and institutional trust limited their perceived accessibility. This divergence between availability and usability highlights the need for trust-building and structural reform.

Licensing-related concerns also emerged as a significant barrier, particularly among resident physicians and participants who discussed prior experiences utilizing mental health care resources. These apprehensions are not unfounded; historically, state licensure applications have included questions about mental health diagnoses and treatment history, often grouped with disciplinary or criminal inquiries. [13,14] In the 1993 case of Medical Society of New Jersey v Jacobs, the court ruled that questions pertaining to mental-health diagnosis or treatment history violated the Americans with Disabilities Act by discriminating against physicians with disabilities. However, substantive reform to policies remained inconsistent, evidenced in a 2007 survey of state medical licensing board directors. [33] Findings suggested approximaely one-third of respondents indicated a physician could be justifiably sanctioned for reporting a mental health diagnosis. [33]

More recently, a significant push for policy reform led by a coalition of leading healthcare organizations including the Dr. Lorna Breen Heroes’ Foundation, Harvard T.H. Chan School of Public Health, Thrive Global, and CAA Foundation, has driven substantial change. As of September 19, 2025, 40 medical licensure boards (initial and renewal MD and DO applications) and 6 nursing licensure boards (APRN applications) have verified that intrusive mental health questions are not included on their licensing applications. [34] Despite these advances, the persistence of variability across jurisdictions means that many clinicians remain uncertain about the implications of disclosure, sustaining real regulatory risk and reinforcing fear. This suggests that barriers are both perceived and reflective of regulatory risk, underscoring the need for continued advocacy and uniform policy adoption to mitigate deterrents to help-seeking.

Relatedly, a novel and important finding was the lack of trust in institutional confidentiality, described by participants as a major deterrent to help-seeking. This distrust reflects concerns that information shared through employer-sponsored programs could be accessed by administrators or influence professional standing. Such perceptions may undermine the effectiveness of internal wellness initiatives, even when resources are technically available. Practical implications for intervention design include prioritizing confidentiality safeguards, clearly communicating privacy protections, and considering partnerships with external mental health providers to reduce perceived institutional control. These strategies are critical for rebuilding trust and ensuring that clinicians feel safe accessing support.

Finally, participants shared concerns about institutional financial goals as a source of stress, indicating they feel as though their well-being was secondary to productivity metrics. While prior studies have identified administrative burdens and resource constraints as stressors [32], a novel contribution of this study is the identification of perceptions that institutional priorities, particularly productivity and revenue generation, undermine clinician well-being. Participants described constant pressure to meet performance metrics, such as patient throughput and documentation times, which they perceived as prioritizing efficiency over provider health. These perceptions may interact with stigma by reinforcing norms that equate help-seeking with inefficiency or reduced productivity, thereby discouraging disclosure. Furthermore, institutional emphasis on productivity may exacerbate structural barriers, including lack of time and distrust in organizational confidentiality, as clinicians fear that seeking care could signal reduced capacity to meet institutional expectations. Addressing these concerns requires organizational strategies that explicitly integrate clinician well-being into performance frameworks, signaling that mental health support is compatible with institutional success.

Together, findings from the present study underscore the multifaceted nature of barriers experienced by clinicians in emergency medicine accessing mental health care resources. By integrating quantitative and qualitative data, and situating findings within existing literature and stigma theory, the present study highlights how institutional, cultural, and regulatory factors intersect to shape clinician perceptions and experiences. The results not only reinforce known challenges such as stigma, time constraints, and licensure-related apprehensions but also reveal emerging concerns, including the impact of institutional financial priorities. These insights underscore the need for multi-level interventions that go beyond individual resilience to address systemic and structural reforms. Future research and policy efforts should prioritize creating supportive environments that normalize help-seeking, protect confidentiality, and align institutional goals with clinician and patient well-being. Additionally, research should continue to explore how intersecting identities (e.g., gender, career stage) and organizational contexts shape provider experiences and outcomes.

### Limitations

Although this study used mixed methods, combining a quantitative survey and qualitative interview, to obtain detailed information about emergency department clinicians, there are several notable limitations to the work in relation to the study sample, measurements used, and generalizability of findings. First, the sample size was modest, with 43 survey respondents and 16 interview participants drawn from a single academic emergency department. Although the response rate was reasonable for clinician surveys, the small sample reduces statistical power and limits the ability to detect nuanced differences across subgroups (e.g., clinician types). Second, the sample was predominantly White, cisgender, and heterosexual, which constrains the generalizability of findings to more diverse clinician populations and institutional contexts. There remains a notable gap in the research on the help seeking behaviors of providers belonging to underrepresented racial, ethnic, and sexual minority groups. Future research could prioritize multi-site recruitment and oversampling under-represented groups to capture intersectional experiences.

Third, although validated instruments (PHQ-9 and SSOSH) were used for key constructs, other measures, such as the “Barriers to Seeking Mental Health Care” and “Occupational Stressors and Experiences” lists, were developed for this study and evaluated primarily through face validity and pilot testing. These tools lack formal psychometric validation, and findings should be considered preliminary until replicated with validated measures.

Fourth, the participant sample within phase 1 (i.e., the survey) was about equally split between males and females whereas phase 2 (i.e., the qualitative interview) had a higher proportion of women and more experienced providers. This may have limited how well we qualitatively captured male provider experiences and experiences of those providers who entered the field more recently.

### Implications for practice

The findings of this study, although preliminary, identify several issues that may hold relevance for healthcare practice. First, they highlight the potentially negative impact on clinicians resultant from the structure of state licensing applications requiring mental health care treatment information. Licensing bodies and institutions should consider revising licensure-related policies to ensure mental health questions focus only on current impairment, thereby reducing concerns related to disclosure. Second, these findings highlight the potential benefit from operationally tailored support programs for healthcare providers. Institutions could develop and implement structured mental health support programs specifically designed for emergency department clinicians, such as on-site counseling services, peer support groups, and access to confidential telehealth options. Careful consideration should be made to implementing confidentiality safeguards and communicating these clearly to rebuild trust among clinicians. Finally, these findings suggest that a healthier workforce leads to better patient outcomes and reduced turnover; as such, there is potential for healthcare systems to benefit by operationalizing this priority (e.g., by embedding clinician well-being metrics into performance dashboards, allocating dedicated resources for wellness programs, and holding leadership accountable for integrating mental health support into organizational quality-of-care strategies).
